# Lack of the Receptor for Advanced Glycation End-Products Attenuates *E. coli* Pneumonia in Mice

**DOI:** 10.1371/journal.pone.0020132

**Published:** 2011-05-23

**Authors:** Lasse Ramsgaard, Judson M. Englert, Michelle L. Manni, Pavle S. Milutinovic, Julia Gefter, Jacob Tobolewski, Lauren Crum, Gina M. Coudriet, Jon Piganelli, Ruben Zamora, Yoram Vodovotz, Jan J. Enghild, Tim D. Oury

**Affiliations:** 1 Department of Pathology, University of Pittsburgh School of Medicine, Pittsburgh, Pennsylvania, United States of America; 2 Division of Immunogenetics, Department of Pediatrics, Children's Hospital of Pittsburgh, Pittsburgh, Pennsylvania, United States of America; 3 Department of Surgery and the Center for Inflammation and Regenerative Remodeling, McGowan Institute for Regenerative Medicine, University of Pittsburgh, Pittsburgh, Pennsylvania, United States of America; 4 Department of Molecular Biology, Center for Insoluble Protein Structures (inSPIN) and Interdisciplinary Nanoscience Center (iNANO), University of Aarhus, Aarhus, Denmark; Hannover School of Medicine, Germany

## Abstract

**Background:**

The receptor for advanced glycation end-products (RAGE) has been suggested to modulate lung injury in models of acute pulmonary inflammation. To study this further, model systems utilizing wild type and RAGE knockout (KO) mice were used to determine the role of RAGE signaling in lipopolysaccharide (LPS) and E. coli induced acute pulmonary inflammation. The effect of intraperitoneal (i.p.) and intratracheal (i.t.) administration of mouse soluble RAGE on *E. coli* injury was also investigated.

**Methodology/Principal Findings:**

C57BL/6 wild type and RAGE KO mice received an i.t. instillation of LPS, *E. coli*, or vehicle control. Some groups also received i.p. or i.t. administration of mouse soluble RAGE. After 24 hours, the role of RAGE expression on inflammation was assessed by comparing responses in wild type and RAGE KO. RAGE protein levels decreased in wild type lung homogenates after treatment with either LPS or bacteria. In addition, soluble RAGE and HMGB1 increased in the BALF after *E. coli* instillation. RAGE KO mice challenged with LPS had the same degree of inflammation as wild type mice. However, when challenged with *E. coli*, RAGE KO mice had significantly less inflammation when compared to wild type mice. Most cytokine levels were lower in the BALF of RAGE KO mice compared to wild type mice after *E. coli* injury, while only monocyte chemotactic protein-1, MCP-1, was lower after LPS challenge. Neither i.p. nor i.t. administration of mouse soluble RAGE attenuated the severity of *E. coli* injury in wild type mice.

**Conclusions/Significance:**

Lack of RAGE in the lung does not protect against LPS induced acute pulmonary inflammation, but attenuates injury following live *E. coli* challenge. These findings suggest that RAGE mediates responses to *E. coli*-associated pathogen-associated molecular pattern molecules other than LPS or other bacterial specific signaling responses. Soluble RAGE treatment had no effect on inflammation.

## Introduction

Acute lung injury and acute respiratory distress syndrome (ARDS) are caused by various factors such as aspiration, hyperoxia, bacterial pneumonia, and sepsis. Damage to the alveolar epithelium leads to an inflammatory response mediated by release of inflammatory cytokines from activated macrophages. The inflammatory cytokines recruit inflammatory cells from the blood and increase the permeability of the alveolar-capillary membrane. As a result, proteinaceous fluid accumulates in the lung, leading to decreased pulmonary function [1].

The receptor for advanced glycation end-products (RAGE) was originally purified from bovine lung and was shown to have affinity towards advanced glycation end-products [2]. RAGE expression has since been found to be very high in the normal lung compared to low or no expression in other tissues. Furthermore, in contrast to most organs where RAGE expression increases with disease [3], [4]; RAGE levels decrease significantly in the injured lung [5]–[8]. RAGE is primarily expressed on epithelial type I cells [9], [10] and has in recent years been implicated in various forms of fibrosis and diabetic vascular disease [5], [7], [8], [11]–[13] and more recently in acute lung injury and sepsis [14]–[17].

A recent study found that i.p. administration of soluble RAGE attenuated LPS-induced acute lung injury in mice [16]. In addition, soluble RAGE has been shown to accumulate in the BALF and sera of patients with acute lung injury and is thought to be a marker of alveolar type I cell injury [18]–[21]. However, the effect of these injuries on overall pulmonary RAGE expression has not been determined. As RAGE ligands are not specific for RAGE, the finding that soluble RAGE protected against LPS-induced lung injury may be due to blocking membrane RAGE signaling or by preventing RAGE ligands from binding other receptors. In fact, the inflammatory response against LPS is mediated by recognition of the LPS molecule by TLR4 primarily on macrophages. Binding of LPS to TLR4 leads to an up regulation of pro-inflammatory cytokines [22]. One of these cytokines, high mobility group box 1 (HMGB1), is a damage-associated molecular pattern molecule (DAMP) that binds to and activates TLR2- and -4 [23], [24], but is also a ligand for RAGE [2], [25]. Activation of RAGE leads to activation of nuclear factor kappa B (NF-κB), which in turn leads to a further increase in transcription of pro-inflammatory cytokines [26], [27] and a positive feedback loop that results in sustained inflammation.

In the current study, both a LPS and a bacterial (*E. coli*) pneumonia model were used to study the role of RAGE in acute pulmonary inflammation. LPS is a pyrogenic molecule present in the cell wall of Gram-negative bacteria such as *E. coli* and belongs to the group of pathogen-associated molecular patterns, which signals danger via receptors such as the Toll-like receptors (TLRs) and other pattern recognition receptors [28]. In addition, administration of purified mouse soluble RAGE to wild type mice challenged with *E. coli* was performed to determine if this could offer any therapeutic effect and to also distinguish between the effects of membrane and soluble RAGE.

## Materials and Methods

### Ethics Statement

All animal experiments were reviewed and approved by the Institutional Animal Care and Use Committee at the University of Pittsburgh. Animals were given free access to food and water and were cared for according to guidelines set by the American Association for Laboratory Animal Care.

### Animal experiments

The constitutive global RAGE KO strain was generated on a C57BL/6 background [29]. C57BL/6 wild type mice were purchased from Taconic (Germantown, NY). Only male mice were used in the experiments.

LPS serotype 055:B5 (Sigma-Aldrich, St. Louis, MO) was diluted in PBS. Mice received i.t. instillations of 75 µg LPS in a total volume of 75 µL. For *E. coli* (ATCC, Manassas, VA, #25922) challenge, mice received i.t. instillation of 1×10^6^ CFU's in 50 µL of PBS. For i.p. treatment with mouse soluble RAGE mice received 100 µg right before *E. coli* instillation, control mice in the same experiment all received an equal volume of PBS i.p. The choice of a single soluble RAGE injection was based on prior studies showing that i.v. administered recombinant rat soluble RAGE has a half-life of 26 hours in rats [30]. In addition, this dose of soluble RAGE was previously shown to be effective in LPS induced lung injury [16]. Mice receiving both *E. coli* and soluble RAGE i.t. were given 50 µg of soluble RAGE and 1×10^6^ CFU's in 50 µL of PBS.

Mouse soluble RAGE was purified and endotoxin removed as previously described [31]. Biological activity of the purified soluble RAGE was confirmed by showing concentration dependent binding to HMGB1 (A RAGE ligand; Sigma-Aldrich) in an ELISA like setup as previously described [10], [32] (not illustrated). Protein in PBS and PBS used for i.p. and i.t. injections/instillations had endotoxin levels below an administration of 5 EU/kg mouse. Endotoxin levels were determined using the limulus amebocyte lysate single test vial assay (Associates of Cape Cod, East Falmouth, MA).

### Inflammatory cells

Mice were sacrificed by an overdose of pentobarbital sodium (Ovation Pharmaceuticals Inc., Deerfield, IL) and the lungs were lavaged with a single instillation of 800 µL normal saline. Recovery of BALF was consistently above 75%. BALF cells were counted using a Z1 Coulter Particle Counter (Beckman Coulter Inc., Fullerton, CA) and 30,000 cells were transferred to glass slides using a Shandon Cytospin 4 (Thermo Electron Corporation, Pittsburgh, PA) at 750 rpm for 5 min. After 2 days of drying, cells were stained using the Diff-Quick stain (Dade Behring Inc., Newark, DE), and cell differentials were determined by counting 200 cells.

### Protein concentration in BALF

Protein concentration in undiluted BALF was measured in triplicate by a standard endpoint measurement at 495 nm using Bradford reagent with a bovine serum albumin standard curve (Thermo Scientific, Rockford, IL).

### Cytokine profile of BALF

IL-1β, IL-6, IL-12(p70), KC, MCP-1, MIP-1α, and TNF-α concentrations were measured in BALF samples using an 8 plex Procarta Cytokine Assay Kit – Mouse (Affymetrix, Fremont, CA). The assay was performed in duplicate on 50 µL of BALF using a mouse bodily fluid diluent (Affymetrix) according to manufactures instructions and read on a Luminex™ 100 IS apparatus (MiraiBio, Austin, TX). Some control samples had levels below the limit of detection, labeled ND. For these samples the limit of detection for the individual cytokines was used for the statistical analysis. This was done under the assumption that if a statistical significant difference could be measured using the limit of detection, then the actual value would only strengthen the statistics. BALFs from both strains treated with vehicle control, LPS, and *E. coli* were assayed (6 groups).

### Mouse Lung Slices

Mice were sacrificed by i.p. injection with sodium pentobarbital (35 mg/kg). Lungs were perfused through the heart with cold PBS and removed using aseptic techniques and placed into PBS solution on ice. A McIlwain tissue chopper (Mickle Laboratory Engineering Co. Ltd., United Kingdom) was used to cut the lungs transversely. The slice thickness was chosen to be 0.7 mm which was predicted by Warburg equation: C_1_ = C_0_-A/D*d2/8, where C_1_ is the pO_2_ in the deepest layer of the slice; C_0_ is the pO_2_ at the surface of the slice; A is oxygen uptake of the slice in mL/min/mL of tissue; D – diffusion coefficient in mL of oxygen/cm_2_/min; d – slice thickness in cm [33], [34]. After being cut the slices were placed into a 10 mm dish filled with PBS and washed with agitation. Six pieces were placed into each well of a 6-well tissue culture plate. Slices were maintained in RPMI-1640/HAM'S- F12 medium (Lonza Group Ltd., Switzerland) (1∶1) supplemented with 10% FBS and 1% penicillin-streptomycin. Culture medium containing 500 ng LPS (Serotype 055:B5, Sigma-Aldrich, St. Louis, MO) were applied 1 hour after seeding. After 24 hours the slices were harvested in TRI-Reagent, total RNA was extracted as directed by the manufacturer (Molecular Research Center, Cincinnati, OH) and used for Luminex RNA assay for 5 known inflammatory cytokines (IL-1α, IL-6, IL-1β, TNF-α and MCP-1). All experiments were performed in triplicate.

### Histology

Lungs used for histology were inflation fixed with 800 µL of 10% neutral buffered formalin for 8 min and then paraffin embedded. This was done by placing a lavage needle in a small incision in the exposed trachea. A suture was used to tie around the needle and trachea in order to make a tight seal. After the 8 minutes the lung was removed and placed in neutral buffered formalin for a minimum of 6 hours, followed by 95% ethanol until paraffin embedding. Five µm sections were H&E stained and the sections were evaluated by light microscopy.

### Myeloperoxidase assay

Lungs were perfused through the right ventricle of the heart with 10 mL of saline to get rid of blood neutrophils, excised, flash frozen in liquid nitrogen and stored at −80°C until use. Lungs were homogenized in 3 mL of homogenizing buffer (50 mM potassium phosphate/10 mM N-ethylmaleimide, pH 6.0). Following centrifugation at 12,000× g for 30 min at 4°C, the pellet was washed twice in 1 mL of homogenizing buffer. The pellet was then resuspended in 1 mL of HTAB buffer (50 mM potassium phosphate/0.5% hexadecyltrimethylammonium bromide, pH 6.0) by sonication. After 3 freeze thaw cycles using liquid nitrogen and a 56°C water bath, the samples were incubated at 56°C for 2 hours. After centrifugation at 12,000× g for 10 min, 10 µL of each sample was mixed with 890 µL of assay buffer (50 mM potassium phosphate/0.167 mg/mL o-dianisidine dihydrochloride/0.0005% H_2_O_2_/0.01% HTAB, pH 6.0) and the absorbance at 460 nm was measured on a Beckman Coulter Spectrometer (Beckman Coulter Inc., Somerset, NJ) every 5 sec. for 2 min. using a kinetic protocol. The myeloperoxidase activity was determined from the slope of the curve and reported as dA/min per lung.

### Lung homogenization

Whole frozen lungs were homogenized into 3 mL of cytoplasmic buffer (10 mM HEPES/10 mM KCl/2 mM MgCl_2_/0.1 mM EDTA/1 mM DTT/1 mM PMSF/10 µM E64/pH 7.8) and incubated on ice for 15 min followed by centrifugation at 12,000× g for 5 min at 4°C. The supernatant was centrifuged again as described above; the supernatant was kept as the soluble/cytoplasmic fraction. The cell pellet from the first centrifugation was washed in 2 mL wash buffer (10 mM HEPES/10 mM KCl/150 mM NaCl/0.1 mM EDTA/1 mM DTT/1 mM PMSF/10 µM E64/pH 7.8) by vortexing and centrifuged as above. The resulting cell pellet was resuspended in 500 µL membrane buffer (Wash buffer with 1% NP-40) vortexed and rotated end-over-end for 30 min at 4°C. After centrifugation as above the supernatant was kept as the membrane fraction. Protein concentration was measured at a 1∶20 dilution as for BALF described above.

### Western blotting

SDS-PAGE was performed with 5–15% gradient gels using the glycine/2-amino-2-methyl-1,3-propanediol/HCl system as previously described [35]. Proteins were transferred onto a PVDF membrane (Millipore, Billerica, MA) and the membrane was blocked in 5% milk in PBS-T. The membrane was incubated with a 1∶5,000 dilution of primary antibody against RAGE generated in rabbit as previously described [5], a 1∶2,000 dilution of rabbit-anti HMGB1 (Abcam, Cambridge, MA) or a 1∶5,000 dilution of mouse-anti β-actin (Sigma-Aldrich). After washing, the membrane was incubated with a 1∶5,000 dilution of a HRP-conjugated donkey-anti-rabbit antibody (GE Healthcare, Buckinghamshire, UK) or a 1∶10,000 dilution of a HRP-conjugated goat-anti-mouse antibody (Jackson ImmunoResearch Laboratories, Inc., West Grove, PA). The membrane was developed using the enhanced chemiluminescent plus reagent (GE Healthcare) for BALF blots or the SuperSignal West Pico Chemiluminescent Substrate (Thermo-Fisher) for lung homogenates and visualized on a KODAK GelLogic 2200 Imaging system (Carestream Health, Rochester, NY). Loading control on lung homogenate blots was performed by stripping the membrane for 1 hr (25 mM Glycine, 1% SDS, pH 2) and reprobing it for β-actin and normalizing band intensity to the β-actin signal.

### Statistical analysis

Data were analyzed using GraphPad Prism 5.0 (GraphPad Software Inc., La Jolla, CA). Experiments involving both wild type and RAGE KO (2 variables; strain and treatment) were analyzed by two-way analysis of variance with a Bonferonni *post hoc* test. Data with one variable were analyzed by a Mann-Whitney test or one-way analysis of variance followed by a Tukey's *post hoc* test. All values are expressed as means (±SEM). p<0.05 was considered significant.

## Results

### Wild type and RAGE KO mice have similar degrees of inflammation after LPS treatment

**Figure 1:**
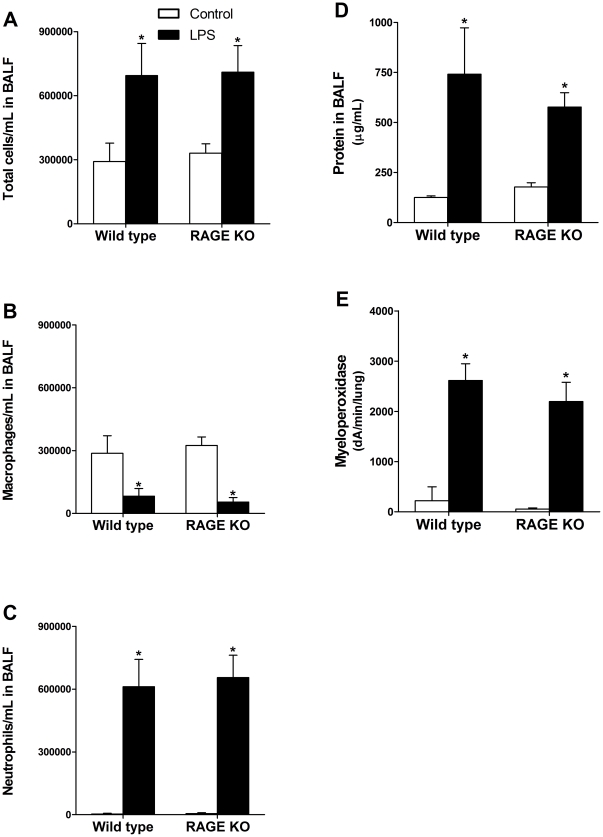
Lack of RAGE does not protect against LPS-induced acute pulmonary inflammation. LPS challenge led to a significant increase in total cells/mL in BALF for both wild type and RAGE KO mice (A). Differential cell counts showed that the increase in cells were mainly neutrophils (B–C). However, there was no significant difference in response between wild type and RAGE KO mice. LPS challenge also led to a significant increase in protein in the BALF over the control treated animals to a similar degree for both wild type and RAGE KO mice (D). Myeloperoxidase activity in the lung was also significantly increased in both strains after LPS injury, but no significant difference was seen between the two (E). Asterisks indicate significant increase/decrease compared to vehicle control for each strain. Data are means (±SEM), analyzed by 2-way ANOVA with a Bonferroni post hoc test (*n of 4–11 per group*).

Twenty-four hours after i.t. challenge with LPS or vehicle control, mice were sacrificed and assayed for various indicators of acute pulmonary inflammation. Injury was assessed by measuring total cell influx and differential cell counts in BALF, as well as protein in BALF and myeloperoxidase activity of whole lung homogenates. While the administration of LPS caused a significant increase in cells and protein in the BALF (Figure 1A, D) compared to the vehicle-treated mice (control), there was no difference between wild type and RAGE KO mice. Differential cell counts illustrate that the major cell type found in the BALF of controls was the macrophage (Figure 1B). In contrast, after LPS challenge, inflammatory cells in the BALF were almost exclusively neutrophils (Figure 1C). Myeloperoxidase activity, a measure of neutrophil infiltration, after injury was also significantly increased over the controls to a similar degree in lung homogenates from both wild type and RAGE KO mice after LPS challenge (Figure 1E).

### RAGE KO mice have less inflammation than wild type mice in response to E. coli challenge

**Figure 2:**
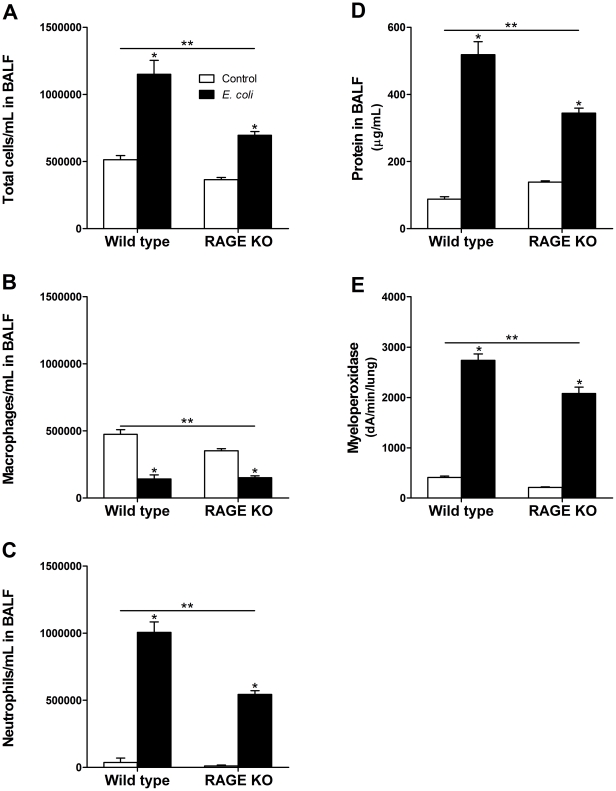
RAGE KO mice get less inflammation than wild type mice after *E. coli* pneumonia. *E. coli* challenged RAGE KO mice had significantly less total cells/mL in BALF compared to wild type mice (A). The lower amount of cells was mainly due to less neutrophils (B, C). Protein in BALF (D) and myeloperoxidase activity of whole lung (E) were also significantly less in RAGE KO than in wild type mice after *E. coli* challenge. * indicate significant increase/decrease compared to vehicle control for each strain. ** indicate significant difference between wild type and RAGE KO mice. Data are mean ±SEM, analyzed by 2-way ANOVA with a Bonferroni post hoc test (*n of 6–8 per group*).

Mice were sacrificed 24 hours after *E. coli* challenge. Both wild type and RAGE KO mice exhibited significantly increased cellularity (total cells/mL) in BALF after *E. coli* injury as compared to control treated mice (Figure 2A). However, in contrast to LPS injured mice, RAGE KO mice had a significantly lower *E. coli*-induced increase in cellularity compared to wild type mice (Figure 2A). Differential cell counts showed that after *E. coli* challenge, RAGE KO mice had a significantly smaller decrease in macrophages and a significantly reduced increase in neutrophils compared to wild type mice (Figure 2B–C). Protein in the BALF was used as a measure of lung permeability and hence injury. The protein concentration in the BALF was significantly increased after *E. coli* injury in both wild type and RAGE KO mice, with the latter having a significantly lower increase than the wild type mice (Figure 2D). Myeloperoxidase analysis also showed significantly less activity in lung homogenates of RAGE KO compared to wild type lungs (Figure 2E) after *E. coli* infection, consistent with the lower levels of neutrophils observed in the RAGE-deficient mice.

### Histology

**Figure 3:**
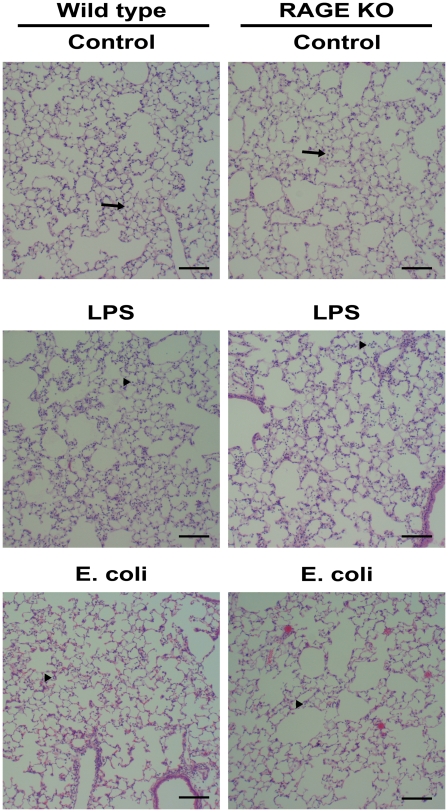
H&E stained lung sections from wild type and RAGE KO control, LPS and *E. coli* treated mice. The first column shows wild type and the second column shows RAGE KO lungs. Macrophages (arrows) and neutrophils (arrowheads) in alveoli are indicated. First row is control treated from both strains. A few macrophages are present in the alveoli of both control treated strains. LPS treatment, second row, led to a significant infiltration of neutrophils into the alveolar space; however no immediate difference in degree of infiltration was noticeable between the strains. The third row shows lung histology from *E. coli* treated mice. *E. coli* treated RAGE KO mice have fewer neutrophils compared to *E. coli* treated wild type mice. Black bars represent 30 µm.

Formalin fixed and paraffin embedded sections of lungs from all groups were stained with H&E (Figure 3). Both wild type and RAGE KO mice treated with vehicle control exhibited no neutrophils and only occasional macrophages in the alveoli. After LPS challenge, both wild type and RAGE KO mice had a substantial, but similar influx of neutrophils into the alveolar space consistent with the findings illustrated in Figure 1. While both wild type and RAGE KO mice also had a substantial influx of neutrophils into the alveolar lumen after injury with *E. coli*, the RAGE KO mice appeared to have a lower degree of neutrophil influx than wild type mice. This is consistent with the findings illustrated in Figure 2.

### RAGE expression decreases in the lungs after injury

**Figure 4:**
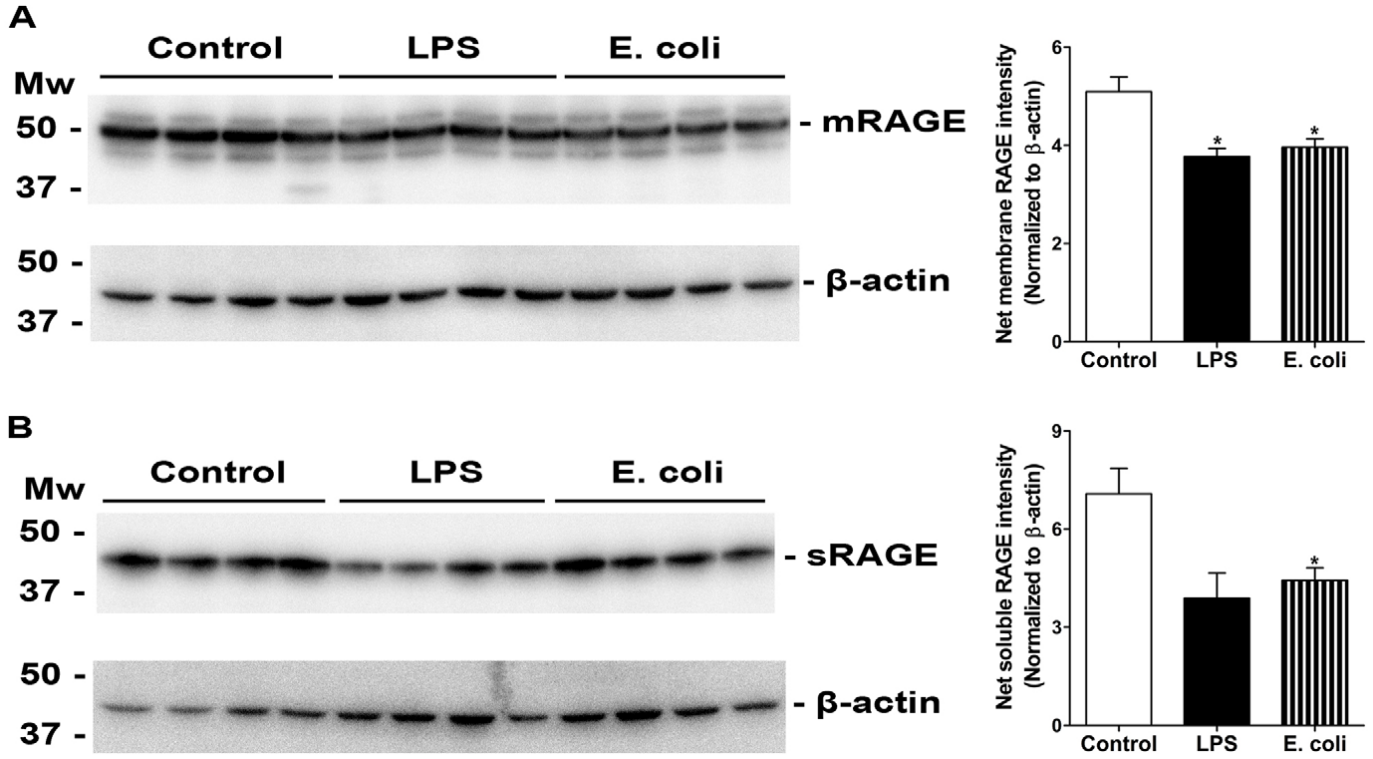
RAGE protein levels are decreased in the lungs after injury. Aliquots of 10 µg of protein from membrane or soluble protein fractions from lung homogenates were assayed for RAGE expression by western blot analysis of RAGE and β-actin. Net intensity of RAGE expression was normalized to β-actin. Membrane RAGE expression was significantly decreased after both LPS and *E. coli* challenge (A). Soluble RAGE expression was significantly decreased after *E. coli* injury, and markedly, but not significantly decreased after LPS injury (p = 0.057 for LPS treatment). Normalized net intensities were analyzed by a Mann-Whitney test. (* p<0.05, *n of 4 mice per group*).

Protein was extracted from control, LPS- and *E. coli*-challenged wild type lungs. RAGE protein levels were assessed by western blot analysis of soluble and membrane protein preparations from lung tissue homogenates using a RAGE-specific primary antibody. Significant decreases in all isoforms of RAGE protein were seen after LPS and *E. coli* injury (Figure 4A–B). Interestingly, in addition to the soluble and membrane RAGE bands (The two lower bands in Figure 4A), an additional upper band of approximately 53 kDa was also detected by western blotting. This upper RAGE band has also been reported elsewhere [6], [36]. This additional RAGE isoform is likely an additional form of membrane RAGE, since it is not present in soluble preparations of whole-lung homogenate (Figure 4B).

### Soluble RAGE and HMGB1 levels in BALF

**Figure 5:**
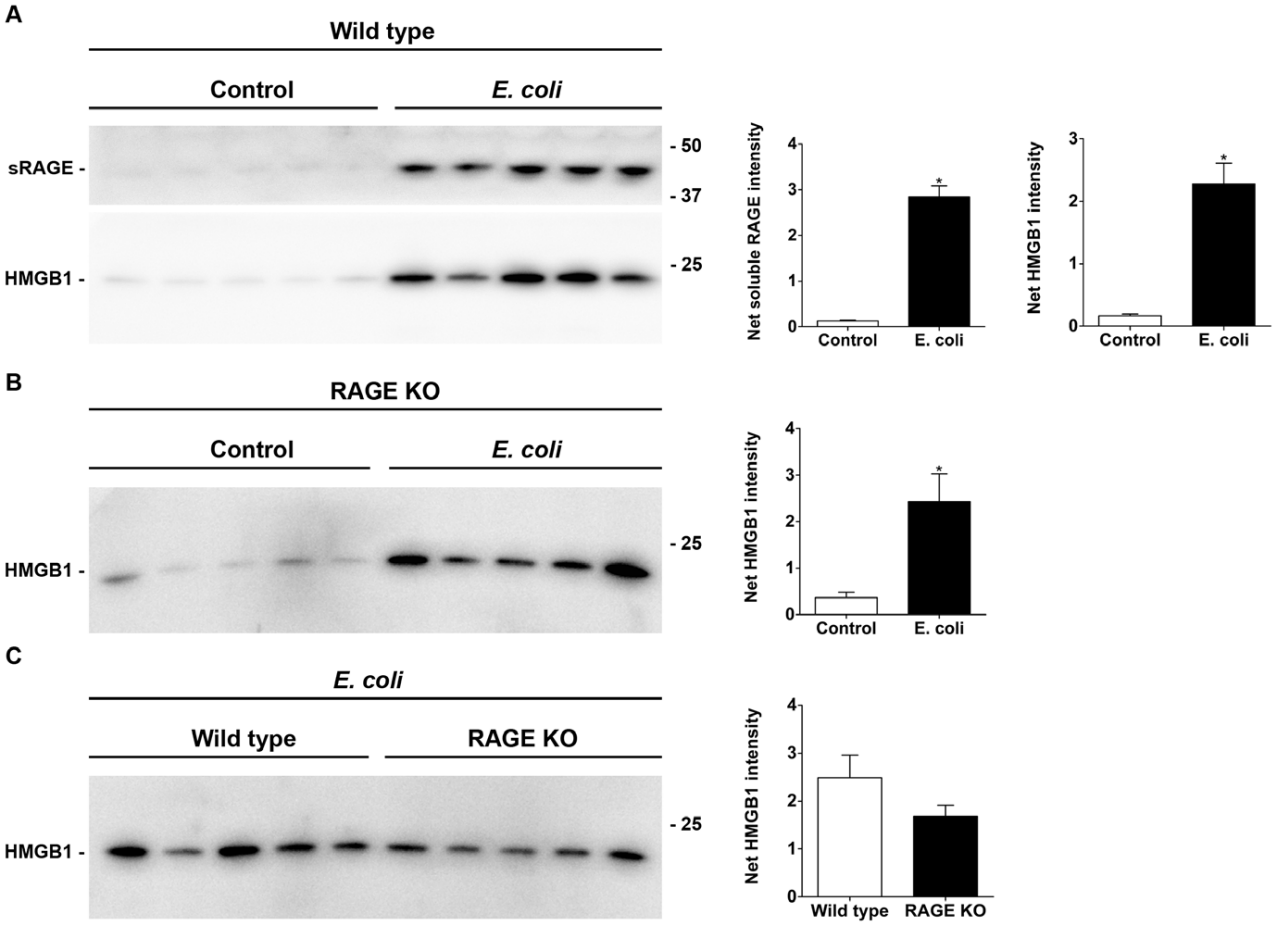
HMGB1 levels are increased in the BALF of wild type and RAGE KO mice after *E. coli* injury. In addition, soluble RAGE is increased in the BALF of wild type mice after *E. coli* injury. Aliquots of 35 µL BALF were loaded per lane. By western blot there is a significant increase in the levels of soluble RAGE and HMGB1 in the BALF from wild type mice (A) and a significant increase of HMGB1 protein in the BALF of RAGE KO mice (B) after *E. coli* injury. Direct comparison of HMGB1 levels between wild type and RAGE KO mice after *E. coli* injury (C) shows a similar level of HMGB1 in BALF from both strains. Comparisons of protein levels were done by band densitometry analysis and analyzed by a Mann-Whitney test (* p<0.05, *n of 5 mice per group*).

The levels of soluble RAGE and HMGB1 in the BALF were investigated by western blot analysis. Since no difference in response to LPS between wild type and RAGE KO mice was seen only the BALF from *E. coli* treated mice were assayed for HMGB1. Soluble RAGE in the BALF increased significantly after *E. coli* challenge compared to the controls in wild type mice (Figure 5A). This finding is consistent with previous studies [16], [21]. Previous reports have found the level of HMGB1 to approximately double in response to acute lung injury as measured by ELISA [16]. By western blot analysis there is a significant increase in the level of HMGB1 (Figure 5A–B) after *E. coli* infection in both wild type and RAGE KO mice. A direct comparison of HMGB1 levels in the BALF from *E. coli* treated wild type and RAGE KO mice showed no significant difference between the two (Figure 5C).

### Cytokine profile of BALF

To identify potential differences in cytokines in the BALF of wild type vs. RAGE KO mice after *E. coli* and LPS injury, BALF samples were assayed for 7 known pro-inflammatory cytokines and chemokines (Table 1). Both LPS and *E. coli* led to a significant increase in all cytokines compared to the controls. After LPS treatment, BALF levels of the chemokine MCP-1 was significantly lower in the RAGE KO mice compared to wild type mice, while all other cytokines were not significantly different between the two strains. These data further support the notion that there are no major differences in the response to LPS between wild type and RAGE KO mice.

**Table 1:** Cytokine profile of BALF from wild type and RAGE KO mice.

	Control		LPS		*E. coli*	
	Wild type	RAGE KO	Wild type	RAGE KO	Wild type	RAGE KO
**IL-1β**	3.9±0.5	3.4±0.2	24.2±1.7	28.5±2.1	16.7±1.1	9.5±0.6[Table-fn nt101] *
**IL-6**	2.9±0.01	2.9±0.02	201.2±41.1	171.3±23.1	134.1±26.6	33.5±5.2[Table-fn nt101] *
**IL-12(p70)**	3.4±0.03	3.4±0.02	4.0±0.1	3.8±0.1	5.0±0.3	3.8±0.1[Table-fn nt101] *
**KC**	ND	ND	323.2±49.6	421.3±51.6	32.6±2.5	24.8±1.5
**TNF-α**	ND	ND	175.1±16.8	159.8±12.4	88.5±12	36.6±3.2[Table-fn nt101] *
**MIP-1α**	ND	ND	78.1±3.5	87.1±6.2	27.0±2.1	16.2±1.4[Table-fn nt101] *
**MCP-1**	7.7±0.02	7.7±0.01	1204±225	602.2±124.8[Table-fn nt101] *	42±13.4	14.7±1.8[Table-fn nt101] *

* = Significant difference between strains.

All cytokines were significantly up regulated after both LPS and *E. coli* treatment compared to the control treated. In addition MCP-1 was the only cytokine significantly less increased in RAGE KO mice compared to wild type after LPS challenge. After *E. coli* treatment all cytokines except KC were increased significantly less in RAGE KO mice compared to wild type mice. The most substantial differences were in IL-6, MCP-1 and TNF-α where levels were 4, 2.9 and 2.4 times higher in wild type BALF compared to RAGE KO BALF.

*significant difference between wild type and RAGE KO (p<0.05).

Data are mean ±SEM, analyzed by 2-way ANOVA with a Bonferroni post hoc test. (*n of 5–7 per group*). Abbreviations IL: interleukin, KC: keratinocyte-derived chemokine, TNF: tumor necrosis factor, MIP: macrophage inflammatory protein, MCP: monocyte chemotactic protein.

In contrast to the findings with LPS, RAGE KO mice had significantly lower levels of all cytokines and chemokines except the chemokine KC when compared to wild type mice in response to *E. coli* infection. This finding is consistent with the other measures of injury that indicate the RAGE KO mice have a lower overall degree of inflammation after *E. coli-*induced pneumonia as compared to wild type mice.

### Lung slices

In order to further investigate the response towards LPS, wild-type or RAGE KO lung slices were treated with vehicle or LPS and then assayed for RNA expression of 5 known inflammatory cytokines and chemokines (Table 2). In contrast to the cytokine analysis of the BALF, there were significant differences in the expression of all cytokines analyzed when this isolated lung system was used. Lung slices from LPS-treated RAGE KO mice exhibited a significantly higher expression of IL-1α, IL-1β and TNF-α and significantly lower expression of IL-6 and MCP-1 compared to LPS treated wild type lung slices.

**Table 2:** Cytokine RNA quantity in lung slices.

	Control		LPS	
	Wild type	RAGE KO	Wild type	RAGE KO
**IL-1α**	0.03±0.01	0.01±0.00	0.11±0.01	0.19±0.02[Table-fn nt105] *
**IL-6**	0.36±0.06	0.08±0.02	1.00±0.09[Table-fn nt105] *	0.4±0.03
**IL-1β**	0.08±0.01	0.07±0.02	0.25±0.02	0.49±0.04[Table-fn nt105] *
**TNF-α**	0.01±0.00	0.01±0.00	0.02±0.1	0.1±0.01[Table-fn nt105] *
**MCP-1**	0.20±0.03	0.05±0.01	2.0±0.38[Table-fn nt105] *	0.63±0.01

* = Significant difference between strains.

IL-1α, IL-1β, and TNF-α levels were all significantly higher in LPS treated RAGE KO lung slices compared to wild type after LPS challenge. IL-6 and MCP-1 were both higher in the LPS treated wild type lung slices compared to LPS treated RAGE KO.

*significant difference between wild type and RAGE KO (p<0.05).

Data are mean ±SEM, analyzed by 2-way ANOVA with a Bonferroni post hoc test. (*n of 3 per group*). Abbreviations IL: interleukin, TNF: tumor necrosis factor, MCP: monocyte chemotactic protein.

### Intraperitoneal administration of soluble RAGE does not protect against E. coli induced acute pulmonary inflammation

**Figure 6:**
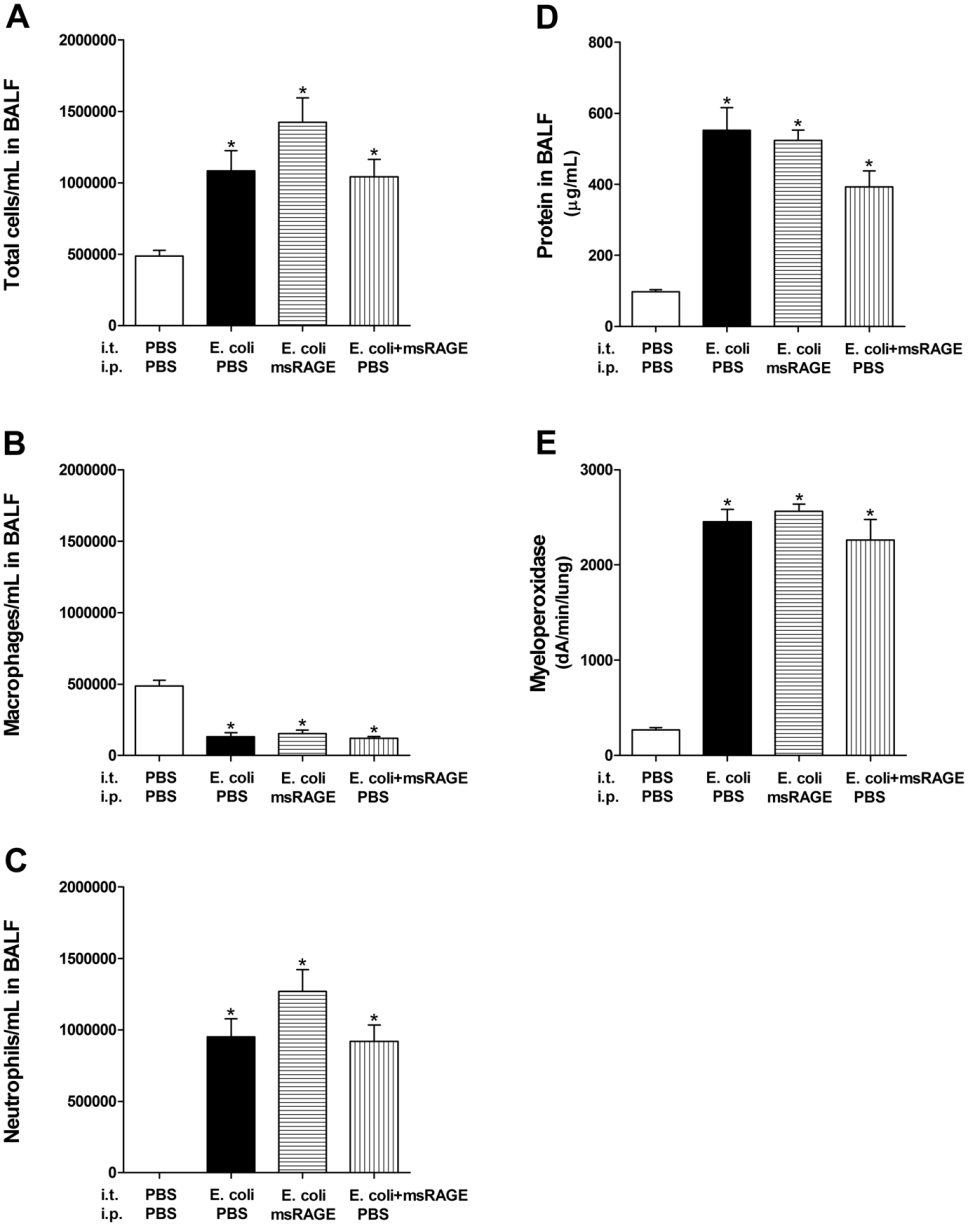
Injection of mouse soluble RAGE (msRAGE) i.p. prior to *E. coli* challenge or i.t. co-treatment with *E. coli* and mouse soluble RAGE did not affect the inflammatory response. Wild type mice only were used for these experiments. Total cells in the BALF and differential cell counts showed no significant difference between *E. coli* mice given vehicle control or i.p. or i.t. msRAGE (A–C). Furthermore, protein in BALF increased the same for mice that received *E. coli* only and the mice that received both *E. coli* and msRAGE i.p. or i.t. (D). Myeloperoxidase activity in lung homogenates was also equally increased over the control for all 3 groups (E). Asterisks indicate significant changes compared to the control. Data are mean ±SEM analyzed by one-way ANOVA followed by a Tukey's post hoc test. (* p<0.05, *n of 5–11 per group*).

As loss of RAGE was found to protect against *E. coli* injury, wild type mice were treated with soluble RAGE to determine if this decoy receptor would offer protection. Mice received 100 µg of purified mouse soluble RAGE by i.p. injection immediately before *E. coli* challenge, and the effect of this treatment was assayed 24 hours later (Figure 6). This dose was based on a previous publication in which the authors administered soluble RAGE to LPS-challenged mice [16]; and this dose has also been found to be effective in other non-pulmonary models of various diseases [37]–[40]. Total cellularity in BALF after *E. coli* challenge with and without mouse soluble RAGE treatment was not significantly different. Mice that received *E. coli* in combination with mouse soluble RAGE had slightly higher, but non-significant, cell counts, which were mainly neutrophils (Figure 6A–C). Protein in BALF and myeloperoxidase activity in lung homogenates were not different when comparing *E. coli*-challenged mice receiving vehicle control vs. mice receiving soluble RAGE i.p. (Figure 6D–E)

### Intratracheal administration of soluble RAGE does not protect against E. coli-induced acute pulmonary inflammation

RAGE is a receptor for HMGB1 [25]. The hypothesized therapeutic effect of soluble RAGE in LPS induced acute lung injury is believed to occur via the ability of soluble RAGE to function as a scavenger receptor for HMGB1 [16], thereby decreasing pro-inflammatory, HMGB1-mediated responses. Because there was no effect of i.p. injection of soluble RAGE on *E. coli* induced inflammation, an additional experiment was performed in which mouse soluble RAGE was instilled i.t. to determine if delivery of this protein directly to the site of injury would ameliorate the inflammatory response. Mice received 50 µg of purified mouse soluble RAGE. This dose was based on the previous finding that one mouse lung contains approximately 75 µg of total soluble RAGE [31]. Hence, 50 µg of mouse soluble RAGE is hypothesized to compensate for any soluble RAGE lost as a result of injury. Figure 6 shows that i.t. instillation of soluble RAGE had no effect on *E. coli*-mediated acute pulmonary inflammation. There was no significant difference in cell concentration in BALF between the mice receiving *E. coli* alone and the mice that also received mouse soluble RAGE (Figure 6A). Differential cell counts revealed an almost identical cell population in mice receiving *E. coli* only and those receiving *E. coli* plus i.t. mouse soluble RAGE (Figure 6B–C).

Protein levels in BALF were slightly lower in the group that received both *E. coli* and i.t. soluble RAGE as compared to *E. coli* alone, but this was not significant (Figure 6D). Myeloperoxidase in total lung homogenate was not significantly different between the two *E. coli* groups (Figure 6E). These results suggest that i.t. instillation of soluble RAGE does not protect against the inflammatory response to pulmonary *E. coli* infection.

## Discussion

In the present study, the role of RAGE in pulmonary acute inflammation was studied by comparing the responses of wild type and RAGE KO mice to LPS and *E. coli*, and by treatment of *E. coli*-infected wild type mice with purified soluble RAGE protein. RAGE KO mice have been studied in bacterial pneumonia previously [14] and soluble RAGE has been administered i.p. in an LPS model of acute lung injury [16]. However, this is the first study in which LPS-induced acute pulmonary inflammation has been evaluated in RAGE KO mice. It is also the first time soluble RAGE has been administered i.t and i.p to evaluate its therapeutic potential in bacterial pneumonia.

RAGE expression is highest in the normal lung compared to all other organs [5], [7], [10], [41]. This seminal finding has given rise to the hypothesis that RAGE must have an important function in the normal lung [5]. Indeed, RAGE is expressed primarily on the basolateral side of type I epithelial cells [9] and has been suggested to be a marker of type I cell injury [18]. In addition, RAGE KO mice have been shown to spontaneously develop pulmonary fibrosis spontaneously with aging and increased fibrosis in response to asbestos injury [5]. In contrast RAGE KO mice are protected against bleomycin-induced pulmonary fibrosis [11], [42], and exhibit a similar degree of fibrosis as wild type mice in a silica model of pulmonary fibrosis [8]. These findings suggest that RAGE indeed has an important function in the lung, but its effects differ depending on the inducing injury. In the current study, RAGE protein expression was found to significantly decrease in lung homogenates in response to two models of acute pulmonary inflammation. This finding is consistent with prior studies examining models of pulmonary fibrosis in which RAGE levels decrease significantly after injury in all models examined [5], [7], [8].

In contrast to the decrease of RAGE protein in the lung homogenates there is an increase in soluble RAGE in the BALF in wild type mice in response to *E. coli* injury. This is consistent with previous studies that showed increased soluble RAGE in the BALF after acute lung injury [16], [21]. The source of the increase in soluble RAGE in the BALF is unclear. However, the increased soluble RAGE in the BALF may be the result of injury to type I epithelial cells and subsequent release/cleavage of membrane RAGE to soluble RAGE. It could also be secondary to soluble RAGE being released from the extracellular matrix following damage to the extracellular matrix structure, or by cleavage of membrane RAGE from viable epithelial cells by proteases released into the inflamed lung. Regardless of the mechanism, this finding correlates well with the decrease in RAGE protein seen in the western blots of the lung homogenates.

RAGE KO mice have also been used to study the role of RAGE in a *Streptococcus* pneumonia model, *E. coli* abdominal sepsis, and a cecal ligation and puncture (CLP) model of polymicrobial sepsis [14], [15], [43]. The Gram-positive bacterium *Streptococcus pneumoniae* was used to induce pneumonia by i.t. instillation [14] and the RAGE KO survival rate was found to be 19% after 8 days, whereas all wild type mice had died by day 4. That study also found the overall inflammatory response to be lower in the RAGE KO mice, and that peritoneal macrophages from RAGE KO mice exhibited increased bacterial killing *in vitro*. However, it was unclear if the increase in bacterial killing by RAGE KO peritoneal macrophages was due to actual increased killing potential or if fewer bacteria were engulfed by the macrophages.

RAGE has also been shown to be an important mediator of antibacterial activity. In a mouse model of *E. coli-*induced abdominal sepsis, RAGE KO mice exhibited impaired antibacterial function compared to wild type mice [15]. In contrast, in a CLP model of polymicrobial sepsis, RAGE KO mice demonstrated better survival than wild type mice. Furthermore, continuous administration of soluble RAGE to wild type mice after CLP improved survival by approximately 30% as compared to mice treated with vehicle control [43]. These diverse studies point to the importance of RAGE in inflammatory responses induced by bacteria and LPS.

The current analysis of mice at a 24 hour time point did not reveal any difference in inflammation between wild type and RAGE KO mice treated with LPS, but did show that RAGE KO mice were significantly protected compared to wild type mice when challenged with live *E. coli*. To further investigate what might explain this difference, the cytokine/chemokine profile was analyzed in BALF from treated mice. Since the concentration of MCP-1 in the BALF after LPS challenge was nearly 2-fold higher in the wild type mice compared to RAGE KO mice, one would expect more macrophages in the BALF of wild type mice. However, Figure 1B illustrates that while macrophage levels are slightly elevated in the wild type mice compared to RAGE KO mice, there is no significant difference between the two.

After *E. coli* challenge, the cytokines IL-6 and TNF-α as well as the chemokine MCP-1 were most substantially increased in wild type as compared to RAGE KO mice. IL-6 is secreted by macrophages, and is substantially increased in the BALF of humans in the early phase ARDS. The expression of IL-6 is partly regulated by TNF-α and IL-1β [44]. TNF-α is secreted from stimulated monocytes and is up regulated by IL-1 and LPS [45]. MCP-1 recruits monocytes, but not neutrophils from the bloodstream to sites of tissue injury and inflammation [46]. Furthermore, TNF-α signaling activates NF-κB, which drives the expression of numerous pro-inflammatory cytokines and chemokines, including MCP-1 [45]. While at this point the exact role of RAGE in the inflammatory response to live *E. coli* is unknown, the decreased levels of cytokines certainly point to an overall decrease in the inflammatory response and cascade of pro-inflammatory cytokine signaling in the RAGE KO mice.

These data are partly supported by the lung slice analysis, in that MCP-1 was also significantly higher after LPS treatment in wild type compared to RAGE KO lung slices. A much more profound difference in the response to LPS was observed in isolated lung slices as compared to the BALF from the live mouse. This difference is mostly likely explained by the much more sophisticated response towards LPS in the live organism compared to an isolated system, and may imply that RAGE is involved in LPS-induced expression of cytokines that remain tissue-associated, vs. cytokines that are secreted into the BALF. Alternatively, RAGE may modulate cytokine/chemokine mRNA expression in a manner that does not translate to the protein level.

RAGE is a receptor for HMGB1, which is an important mediator of the inflammatory response in acute lung injury [47], [48] and sepsis [43]. In the present study, HMGB1 levels in the BALF from *E. coli-*infected wild type and RAGE KO mice were not significantly different between the two strains. The lack of RAGE in RAGE KO mice might be predicted to lower inflammation, due to lack of RAGE-mediated inflammatory response induced by HMGB1. However, this prediction is complicated by the presence of soluble RAGE in wild type mice that can scavenge HMGB1, thereby yielding the same overall response as that seen in RAGE KO mice. The finding that soluble RAGE treatment did not protect against *E. coli* infection suggests that membrane RAGE signaling is of primary importance in this model. Alternatively, this finding may suggest that soluble RAGE treatment, regardless of delivery method, is not reaching the correct compartment (i.e. basement membrane side of type 1 epithelial cells) to be effective.

A recent study found that i.p. administration of recombinant mouse soluble RAGE one hour after i.t. LPS challenge ameliorated the inflammatory response [16]. This finding led to the hypothesis that soluble RAGE could potentially be used as a therapy for patients hospitalized with acute lung injury or ARDS. The therapeutic action of soluble RAGE was suggested to be due to its ability to sequester HMGB1 and thereby function as a decoy molecule, preventing HMGB1 signaling via other pattern recognition receptors. To investigate this possibility in the current study, wild type mice were treated with soluble RAGE purified directly from mouse lungs. However, in contrast to the previous study on the effects of soluble RAGE on LPS-induced inflammatory responses [16], the current experiments suggest that exogenously administered soluble RAGE did not have any therapeutic, protective or ameliorative effect on *E. coli* pneumonia.

Additional studies are necessary in order to determine if the difference in effects in these studies is due to the source of soluble RAGE, the experimental paradigm used, or other factors. As shown in a study investigating the effect of simultaneous administration of LPS i.t. and i.p., removal of LPS from injectable agents is very important [49]. A 30% decrease in neutrophil infiltration of the lung was observed in rats that received i.p. injections of LPS in addition to i.t. LPS compared to rats receiving only i.t. LPS. This finding suggests that any observed effect of injecting soluble RAGE, if not LPS-free, could be due to a diversion of the inflammatory response to the site of soluble RAGE administration. The soluble RAGE used in the current study consistently tested well below the FDA guidelines of 5 EU/kg/day. The lack of a direct increase in neutrophil levels in the BALF after i.t. instillation of soluble RAGE only further confirms the absence of LPS contamination. While a recent study found that soluble RAGE can act as a chemotactic stimulus for neutrophils both *in vivo* and *in vitro* (34), the present study illustrates that when mouse soluble RAGE is administered by i.t. instillation without other treatments, it does not appear to induce an increase of neutrophils in the BALF. Instead, it led to a slight non-significant increase in macrophages (not illustrated).

The data from the *Streptococcus pneumoniae* study [14], taken together with the data from the current study, strongly suggests that RAGE is involved in the pulmonary inflammatory response to live bacteria. It is important to note that *Streptococcus pneumoniae* is a Gram-positive bacterium, and therefore does not express LPS but instead expresses lipoteichoic acid in its cell wall. *E. coli*, on the other hand, is a Gram-negative bacterium and expresses LPS on its cell wall. The inflammatory responses of the RAGE KO mice were significantly lower when compared to that of wild type mice in both of these models of live bacterial pneumonia. In contrast, LPS-treated RAGE KO mice did not exhibit different inflammatory responses when compared to wild type mice. Hence, the role of RAGE in mediating pulmonary inflammation in response to bacterial infection seems to be independent of LPS.

In summary, the studies described herein with wild type versus RAGE KO mice suggest that RAGE does contribute to the inflammatory response within the first 24 hours after *E. coli* infection. Furthermore, the effect of treatment with various doses and sites of delivery of mouse soluble RAGE showed no protective effects against *E. coli*-induced inflammation. The true role of RAGE will require additional studies, especially to determine if the effects that have been reported using RAGE KO mice are due to changes in the expression of other molecules that compensate for the lack of RAGE in these constitutive knockout mice.
